# Stroke Is Predicted by Low Visuospatial in Relation to Other Intellectual Abilities and Coronary Heart Disease by Low General Intelligence

**DOI:** 10.1371/journal.pone.0046841

**Published:** 2012-11-07

**Authors:** Eero Kajantie, Katri Räikkönen, Markus Henriksson, Jukka T. Leskinen, Tom Forsén, Kati Heinonen, Anu-Katriina Pesonen, Clive Osmond, David J. P. Barker, Johan G. Eriksson

**Affiliations:** 1 National Institute for Health and Welfare, Helsinki, Finland; 2 Children's Hospital, Helsinki University Central Hospital and University of Helsinki, Helsinki, Finland; 3 University of Helsinki, Department of Behavioural Sciences, Helsinki, Finland; 4 National Supervisory Authority for Welfare and Health, Helsinki, Finland; 5 Centre of Military Medicine, Finnish Defence Forces, Lahti, Finland; 6 National Defence College, Finnish Defence Forces, Tuusula, Finland; 7 Vasa Central Hospital, Vasa, Finland; 8 Department of General Practice and Primary Health Care, University of Helsinki, Helsinki, Finland; 9 Vasa Health Care Centre, Vasa, Finland; 10 MRC Epidemiology Resource Centre, University of Southampton, Southampton, United Kingsom; 11 Heart Research Center, Oregon Health and Science University, Portland, Oregon, United States of America; 12 Chair of Fetal Programming, King Saud University, Riad, Saudi Arabia; 13 Unit of General Practice, Helsinki University Central Hospital, Helsinki, Finland; 14 Folkhälsan Research Centre, Helsinki, Finland; Royal Holloway, University of London, United Kingdom

## Abstract

**Background:**

Low intellectual ability is associated with an increased risk of coronary heart disease and stroke. Most studies have used a general intelligence score. We studied whether three different subscores of intellectual ability predict these disorders.

**Methods:**

We studied 2,786 men, born between 1934 and 1944 in Helsinki, Finland, who as conscripts at age 20 underwent an intellectual ability test comprising verbal, visuospatial (analogous to Raven's progressive matrices) and arithmetic reasoning subtests. We ascertained the later occurrence of coronary heart disease and stroke from validated national hospital discharge and death registers.

**Results:**

281 men (10.1%) had experienced a coronary heart disease event and 131 (4.7%) a stroke event. Coronary heart disease was predicted by low scores in all subtests, hazard ratios for each standard deviation (SD) lower score ranging from 1.21 to 1.30 (confidence intervals 1.08 to 1.46). Stroke was predicted by a low visuospatial reasoning score, the corresponding hazard ratio being 1.23 (95% confidence interval 1.04 to 1.46), adjusted for year and age at testing. Adjusted in addition for the two other scores, the hazard ratio was 1.40 (1.10 to 1.79). This hazard ratio was little affected by adjustment for socioeconomic status in childhood and adult life, whereas the same adjustments attenuated the associations between intellectual ability and coronary heart disease. The associations with stroke were also unchanged when adjusted for systolic blood pressure at 20 years and reimbursement for adult antihypertensive medication.

**Conclusions:**

Stroke is predicted by low visuospatial reasoning scores in relation to scores in the two other subtests. This association may be mediated by common underlying causes such as impaired brain development, rather than by mechanisms associated with risk factors shared by stroke and coronary heart disease, such as socio-economic status, hypertension and atherosclerosis.

## Introduction

Children and young adults who gain lower scores in intellectual ability tests have shorter life expectancy [1], [2], [3], [4], [5], [6], [7] and a higher risk of coronary heart disease in later life [8], [9], [10], [11], [12], [13], [14]. Recent studies suggest that they also have a higher risk of stroke [8], [10], [14], [15]. In some studies this association is at least as powerful as that for coronary heart disease, whereas other studies have shown a weaker association [8] or none at all [9]. Whether the predictive ability of low intellectual ability for coronary heart disease is different from that for stroke is potentially important because such differences may help to understand the mechanisms that link intellectual ability with disease.

Most previous studies have used a general measure of intellectual ability. While the predictive validity of the concept of “general intelligence” is well established, intelligence consists of different abilities which may in part have different developmental origins [16], different neural correlates [17] and different predictive value on educational and occupational outcomes [18], [19]. We studied whether verbal, visuospatial and arithmetic abilities, tested during compulsory military service in young adulthood, predict coronary heart disease and stroke in men now aged over 60 years.

## Methods

### Ethics statement

The Helsinki Birth Cohort Study has been approved by the Ethics Committee of the National Public Health Institute, and military service data were linked with permission from the Finnish Defence Command. As only data from these registers were used, no individual consent was required.

### Subjects

The study population comes from the Helsinki Birth Cohort which includes 8760 subjects (4630 men and 4130 women) who were born at Helsinki University Central Hospital between 1934 and 1944 and who attended child welfare clinics in Helsinki. The cohort has been described in detail elsewhere [20]. We obtained permission to link data collected within the Helsinki Birth Cohort Study (HBCS) with data from compulsory military service for these 4630 men, of whom 2786 (60.2%) had intellectual ability test data [1], [16], [21], [22]. The main reason for data unavailability was that the test was gradually introduced in different army regiments from 1955 onwards and conscripts in some regiments did not undergo the test. We have previously reported a detailed non-participation analysis showing that availability of the ability test data was not related to father's occupational status, body size during military service, or maximum achieved education later in life [1].

We defined childhood socio-economic status based on the father's occupation as described [1]. Data for each subject's educational attainment, occupational and marital status were obtained from Statistics Finland for 5-yearly intervals between 1970 and 2000. Maximal educational attainment was grouped into four groups according to the UNESCO classification and maximum achieved occupational status into four groups based on a classification used by Statistics Finland [1]. Data for household taxable income came from Statistics Finland, based on 1980 census. We have previously described the associations between these socio-economic indicators and intellectual ability test scores [1].

### Assessment of intellectual ability at the start of military service

The Finnish Defence Forces Basic Ability Test was developed at the Finnish Defence Forces Education Development Centre. This obligatory test was introduced from 1955 onwards and was given to new recruits during the first 2 weeks of service. It comprises verbal, visuospatial and arithmetic subtests, each composed of 40 multiple-choice questions increasing in difficulty, and has been described elsewhere [16], [23]. Briefly, in the verbal reasoning subtest the subject chooses synonyms, antonyms or words belonging or not belonging to the same category as given words, and similar relationships between two word pairs. In the arithmetic reasoning subtest, the subject completes series of numbers, solves short problems, and computes simple arithmetic operations. The visuospatial reasoning task is analogous to the widely used Raven's Progressive Matrices [24] and consists of a set of matrices with one removed part. The subject is asked to decide which of the given single figures completes the matrix. We collected the test results for each subject from microfilms. The data also included the subject's height, weight and systolic blood pressure. BMI was calculated as kg/m^2^. Systolic blood pressure was missing for 165 men (5.9%), who were excluded from the regression models that included blood pressure.

### Coronary heart disease and stroke

Using the personal identification number of each subject we identified deaths and hospital discharges for coronary heart disease and stroke during 1971–2003 from validated [25], [26], [27], [28] national death and hospital discharge registers; the men were thus 26 to 69 years old during the follow-up. We used the first-ever coronary heart disease event and first-ever stroke event for each subject. For stroke subtypes (hemorrhagic/ischemic), however, we used the type of the first event to avoid uncertainty of diagnosis in subjects who later have a diagnosis of a different stroke subtype. Diagnoses were recorded according to the International Classification of Diseases (ICD). The codes for coronary heart disease were 410–414 in ICD-8 and 9 and I21––I25 in ICD 10 [20]. The codes for stroke were of 430–434 and 436–437 in ICD-8, 430–434 and 436–438 in ICD9; and I60–I69 in ICD-10. For thrombotic stroke, they were 432–434 and 436–437 in ICD 8, 433–434 and 436 in ICD-9, and I63–I64 in ICD 10; for hemorrhagic stroke 430–431 in ICD-8, 430–432 in ICD-9, and I60–I62 in ICD-10 [25]. We adjusted the analyses for hypertension diagnosed before first outcome event using the Social Insurance Institution's Register of people who receive special reimbursement for medication for the disease [29].

### Data analysis

We converted the ability test results to z scores (SD units) so that the mean z score of the study cohort was zero. Correlation coefficient between the verbal and visuospatial z scores was 0.62, between verbal and arithmetic 0.67, and between visuospatial and arithmetic 0.68. To illustrate the associations with the visuospatial z scores in relation to the two other scores, we included all scores simultaneously in the regression model. To illustrate general intelligence, we calculated the mean of all z scores. We examined these z scores as predictors of coronary heart disease and stroke using Cox proportional hazards model, stratified according to year of birth. As in our previous analyses [1], we adjusted all hazard ratios for year and the subject's age at testing. In line with previous work from this [1] and other cohorts [2], [3], [4], [5], [8], [9], [10], [13], we performed additional adjustments for markers of childhood environment (father's occupational status and the subject's height and BMI at the start of military service) and adult socio-economic status (education, occupational status and income). We tested the proportionality assumption of the Cox model by using a time-varying indicator variable, determining whether the hazard ratio for intellectual ability subscore was the same at different ages. None of these time-varying indicators were statistically significant (p>0.08). We divided the ability test scores into fourths to illustrate the graded nature of the relationships; for trends of hazard ratios, the scores were used as continuous variables. We assessed interactions between the effects of socio-economic status and intellectual ability on stroke by including a product term in the model.

## Results

Of the 2786 men, 281 (10.1%) had experienced a coronary heart disease event. Stroke had been diagnosed in 131 (4.7%) men, of whom 39 had hemorrhagic and 81 thrombotic stroke (one subject had both diagnoses), and 12 an unspecified type of stroke. Mean ages at diagnoses and numbers or deaths are shown in Table 1.

**Table 1:** Characteristics of the 2786 men studied.

**Father's occupational status**		N (%)
Manual worker		1721 (61.8%)
Lower middle		613 (22.0%)
Upper middle		401 (14.4%)
Unknown		51 (1.8%)
**Examination at the start of the military service**		Mean (SD)
Age (y)		20.1 (1.4)
Height (cm)		176.4 (6.2)
Body mass index (kg/m^2^)		22.0 (2.5)
Systolic blood pressure (mmHg)*		124.7 (11.1)
**Intellectual ability scores**		Median (quartiles)
Verbal		27 (20, 33)
Visuospatial		24 (20, 28)
Arithmetic		27 (18, 33)
**Highest education achieved**		N (%)
Basic or less		1064 (38.2%)
Upper secondary		715 (25.7%)
Lower tertiary		607 (21.8%)
Upper tertiary		335 (12.0%)
Unknown		65 (2.3%)
**Highest occupational status achieved**		N (%)
Manual worker		606 (21.8%)
Self-employed		151 (5.4%)
Lower-level employee		724 (26.0%)
Higher-level employee		1203 (43.2%)
Unknown		102 (3.7%)
**Income**		Median (quartiles)
Household income in 1980 (1000 Finnish Marks)		101 (76, 130)
**Special reimbursement for antihypertensive medication** N(%)		
Among subjects who had neither CHD nor stroke	418 (17.4%)	
Among subjects who later had CHD	84 (29.9%)	
Among subjects who later had stroke	32 (23.5%)	
**Numbers of events**	N (%)	
CHD	281 (10.0%)	
Median (quartiles) age at first CHD event (y)	54.3 (48.2, 58.3)	
Death from coronary heart disease	89 (3.2%)	
Stroke	131 (4.7%)	
Median (quartiles) age at first stroke event (y)	56.7 (50.6, 59.6)	
Death from stroke	19 (0.7%)	
Stroke and CHD	27 (1.0%)	

CHD, Coronary heart disease.

* Data missing for 165 men.

### Verbal, visuospatial and arithmetic reasoning subscores as predictors of coronary heart disease and stroke

Table 2 shows that men who had lower scores in intellectual ability tests were more likely to get coronary heart disease. The risk associated with each of the three subtests was similar. Further shown in Table 2 is that the risk of stroke was predicted by low scores in the visuospatial reasoning subtest, whereas verbal and arithmetic reasoning scores were unrelated to the risk of stroke.

**Table 2:** Hazard ratios for coronary heart disease and stroke in men in fourths of the score of each ability test subscale.

	Verbal		Visuospatial		Arithmetic	
	Number of cases/subjects	Hazard ratio (95% CI)	Number of cases/subjects	Hazard ratio (95% CI)	Number of cases/subjects	Hazard ratio (95% CI)
**Coronary heart disease**						
Lowest	93/656	2.2 (1.6 to 3.2)	86/674	1.9 (1.3 to 2.7)	93/697	1.9 (1.3 to 2.8)
2	84/724	1.7 (1.2 to 2.5)	75/631	1.8 (1.2 to 2.6)	71/676	1.5 (1.0 to 2.2)
3	56/664	1.3 (0.9 to 1.9)	77/849	1.3 (0.9 to 2.0)	68/727	1.4 (1.0 to 2.0)
Highest	48/741	1.0 (Referent)	43/632	1.0 (Referent)	46/669	1.0 (Referent)
*Per one unit lower z score*		*1.30 (1.16 to 1.46)*		*1.27 (1.13 to 1.42)*		*1.21 (1.08 to 1.36)*
**Stroke**						
Lowest	30/656	1.0 (0.6 to 1.6)	37/674	1.8 (1.0 to 3.1)	36/697	1.0 (0.6 to 1.7)
2	32/724	1.0 (0.6 to 1.6)	24/631	1.2 (0.7 to 2.2)	34/676	1.0 (0.6 to 1.6)
3	34/664	1.1 (0.7 to 1.8)	50/849	1.9 (1.1 to 3.2)	25/727	0.7 (0.4 to 1.2)
Highest	34/741	1.0 (Referent)	20/632	1.0 (Referent)	34/669	1.0 (Referent)
*Per one unit lower z score*		*1.01 (0.85 to 1.20)*		*1.23 (1.04 to 1.46)*		*1.07 (0.90 to 1.27)*

Analyses are stratified for year of birth and adjusted for age and year at testing.

### Visuospatial reasoning subscores in relation to the other subscores

Table 3 (Model 1) illustrates the specific risk associated with low visuospatial reasoning scores in relation to low scores in the two other subtests. Visuospatial scores adjusted for verbal and arithmetic scores predicted stroke (hazard ratio for one SD lower score 1.40; 95% CI 1.10 to 1.79), with similar hazard ratios for hemorrhagic (1.49; 0.94 to 2.36) and thrombotic (1.31; 0.96 to 1.78) stroke, but did not predict coronary heart disease (1.15; 0.97 to 1.36). However, the mean intellectual ability test score predicted coronary heart disease (1.35; 1.18 to 1.54), but not stroke (1.10; 0.90 to 1.35).

**Table 3:** The effect of adjustment for socio-economic status in childhood and adulthood and blood pressure/hypertension on hazard ratios (95% confidence intervals) for coronary heart disease and stroke, per one unit lower z score.

	Visuospatial score	Visuospatial score adjusted for the other scores	Mean z score of all subtests
**Coronary heart disease**			
Model 1: year and age at testing	1.27 (1.13 to 1.42)	1.15 (0.97 to 1.36)	1.35 (1.18 to 1.54)
Model 2: 1+height and BMI at military service	1.26 (1.12 to 1.41)	1.13 (0.96 to 1.34)	1.34 (1.17 to 1.53)
Model 3: 2+ father's occupational status	1.19 (1.06 to 1.34)	1.12 (0.95 to 1.33)	1.24 (1.08 to 1.43)
Model 4: 3+ adult education	1.09 (0.95 to 1.24)	1.08 (0.91 to 1.29)	1.10 (0.94 to 1.29)
Model 5: 4+ adult occupational status	1.07 (0.94 to 1.23)	1.10 (0.92 to 1.31)	1.07 (0.91 to 1.26)
Model 6: 5+ adult income*	1.11 (0.97 to 1.28)	1.13 (0.94 to 1.35)	1.11 (0.93 to 1.12)
Model 7: 5+ blood pressure/hypertension†	1.06 (0.92 to 1.22)	1.09 (0.91 to 1.31)	1.04 (0.87 to 1.23)
**Stroke**			
Model 1: year and age at testing	1.23 (1.04 to 1.46)	1.40 (1.10 to 1.79)	1.10 (0.90 to 1.35)
Model 2: 1+ height and BMI at military service	1.22 (1.03 to 1.45)	1.40 (1.09 to 1.79)	1.09 (0.89 to 1.33)
Model 3: 2+ father's occupational status	1.23 (1.03 to 1.47)	1.39 (1.09 to 1.79)	1.08 (0.87 to 1.34)
Model 4: 3+ adult education	1.15 (0.95 to 1.40)	1.34 (1.04 to 1.72)	0.97 (0.77 to 1.24)
Model 5: 4+ adult occupational status	1.16 (0.95 to 1.42)	1.33 (1.03 to 1.71)	1.00 (0.78 to 1.29)
Model 6: 5+ adult income*	1.19 (0.97 to 1.47)	1.44 (1.11 to 1.87)	0.97 (0.74 to 1.26)
Model 7: 5+ blood pressure/hypertension†	1.15 (0.93 to 1.41)	1.31 (1.00 to 1.71)	0.98 (0.75 to 1.27)

Analyses are stratified for year of birth.

* Taxable income in 1980 in five categories. Subjects with an event in 1980 or before are excluded from this analysis.

† Systolic blood pressure measured at conscription and reimbursement for medication for hypertension before the outcome event.

### Linearity

The hazard ratios across fourths of each subscore (Table 2) showed no evidence of non-linear associations, and no quadratic trend was observed (p values >0.2). An exception was the association of visuospatial reasoning scores with stroke, which suggested a threshold effect with a lower risk in the highest fourth as compared with the three other fourths. We therefore assessed the linearity of this association in more detail. There was no evidence for a quadratic (p = 0.5), third- (p = 0.1) or fourth-degree (p = 0.5) polynomial association. For visuospatial reasoning scores adjusted for the two other scores, hazard ratios from the lowest to highest fourths were 2.0 (95% CI 1.2 to 3.4), 1.3 (0.8 to 2.3), 1.6 (0.9 to 2.7) and 1.0 (referent).

### Socio-economic status

In line with previous studies in this and other cohorts [1], [2], [3], [4], [5], [8], [9], [10], [13], we assessed markers of socio-economic status in childhood and in adulthood as covariates. These factors were showed stronger correlations with mean intellectual ability than with the visuospatial score adjusted for the two other scores. For men who grew up in manual workers', lower middle or upper middle class families, mean z scores were −0.18, 0.23 and 0.45, respectively (r = 0.28; p<0.0001). Corresponding values for visuospatial z score adjusted for the two other scores were −0.02, 0.02 and 0.04 (r = 0.03; p = 0.4). Mean z scores for people who as adults had achieved basic, upper secondary, lower tertiary and upper tertiary education were −0.41, −0.16, 0.48 and 0.82, respectively (r = 0.51; p<0.0001). Corresponding values for visuospatial reasoning z scores adjusted for the other scores were −0.11, −0.02, 0.16 and 0.02 (r = 0.08; p<0.0001), A similar pattern was seen for adult occupational status (r = 0.50; p<0.0001 for mean score and r = 0.11; p<0.0001 for adjusted visuospatial score) and income (r = 0.26; p<0.0001 and r = 0.04; p<0.06). The associations between mean intellectual ability tests scores and coronary heart disease were slightly attenuated when adjusted for father's occupational status; (Table 3, Model 3). They were further attenuated and approached null when adjusted for educational attainment, occupational status and income in adult life. The association of visuospatial reasoning scores adjusted for verbal and arithmetic scores, with stroke was, however, unaffected even after adjustment for all of these socio-economic indicators (Table 3, Models 4 to 6). Because we have previously shown that the association between intellectual ability and all-cause mortality is stronger among men who grew up in families of low socio-economic status [1], we also tested for interactions with socio-economic status. There was no interaction between the effects of any of the socio-economic indicators in childhood or adult life and intellectual ability mean score or the visuospatial score adjusted for the two other scores, except between adult occupational status and the visuospatial score (p = 0.05): among high officials, hazard ratio per one SD higher score was 0.9; 95% CI 0.6 to 1.4; among the remaining occupational groups combined it was 1.7; 1.2 to 2.3.

### Blood pressure and hypertension

Adjustment for systolic blood pressure measured during military service and reimbursement of medication for hypertension had a negligible effect on the results (Table 3, Model 7).

## Discussion

Our longitudinal follow-up of male military conscripts had two main findings. First, the pattern of low intellectual abilities that predicted stroke was different from the pattern that predicted coronary heart disease, although both disorders share common risk factors including hypertension and atherosclerosis. While lower scores on verbal, visuospatial and arithmetic reasoning subtests were equally strong predictors of coronary heart disease, stroke was predicted by a profile characterized by lower visuospatial reasoning subtest scores in relation to scores in the two other subtests. Second, while adjustment for indicators of socio-economic status in childhood and adulthood explained a major part of the associations with coronary heart disease, the associations with stroke were little affected by adjustment for socio-economic variables.

### Comparisons with previous studies

The association we found between lower premorbid intellectual abilities and higher rates of coronary heart disease is consistent with previous studies [8], [9], [10], [11], [12], [13], [14]. The hazard ratio of 1.35 for one SD lower mean test score was also comparable to previously reported hazard ratios which vary between 1.25 and 1.4, at least before the age of 65 years [8], [9], [10], [11], [12], [13], [14]. Also consistent with previous studies was that these associations were substantially attenuated when adjusted for socio-economic variables in childhood and adulthood, suggesting that low intellectual ability and low socio-economic status are related to coronary heart disease through similar processes.

A novel finding in our study was that stroke was predicted by visuospatial but not by verbal or arithmetic reasoning abilities. Five previous studies have assessed the relationship between premorbid cognitive abilities and stroke: only one includes subscores assessing different abilities. Two of the studies were based on Scottish data and used the Moray House Test [14] or parts thereof [10]. That test contains diverse items with a preponderance of verbal reasoning but produces a total score without differentiating between different abilities [14], [30]. In one of these Scottish studies, the Aberdeen Study with a follow-up until approximately 50 years of age, the test score based on only verbal ability at age 11 years predicted stroke with a hazard ratio of 1.32 for one SD lower scores in men [10]. In the other study based on the whole test battery, the hazard ratio was larger, 1.47, but it was observed only among people diagnosed before 65 years of age when the number of events was relatively small. Among 1.1 million Swedish male conscripts followed up until 28 to 53 years of age, a general intelligence test predicted stroke [15]; transferred to comparable units, the hazard ratio in that study was 1.18 (95% CI 1.16 to 1.21) for one SD lower total IQ score. This was higher than that in the present study although within its confidence interval (1.09; 0.89 to 1.33, for mean score of the three subtests, with corresponding adjustments). In a study of a smaller, partially overlapping Swedish cohort, associations with stroke were similar than in the larger Swedish study and weaker than those for coronary heart disease [8]. Neither of the Swedish studies differentiated between subscores of the test. The only study that did so followed up Danish men who at age 12 underwent a Härnqvist test producing verbal, spatial and inductive intelligence scores. Whereas each of these scores predicted coronary heart disease, none of them predicted stroke [9]. The follow-up, however, was restricted to 47 years. While the comparison of previous studies is difficult be differences in intellectual ability tests used and other cohort characteristics including dissimilar ages during follow-up, it is nevertheless safe to conclude from previous work that stroke is predicted by lower intellectual abilities. Our study adds significantly to this conclusion by demonstrating that visuospatial reasoning, as assessed by a Raven's progressive matrices-type test, may be one of the a core elements in this prediction.

### Possible mechanisms

Mechanisms that link premorbid intellectual abilities with chronic adult disease have been suggested to include adult socioeconomic advantage, a healthier lifestyle, and a theoretical concept of “body system integrity” underlying both intellectual abilities and disease [1], [4], [13]. Consistent with previous studies [8], [11], [13], [14], we found that much of the relationship with coronary heart disease is explained by adult socio-economic indicators. These relationships have been widely discussed elsewhere [8], [11], [13], [14], [31] and we here focus on our findings regarding stroke which we believe reflect the concept of integrity of the body, in particular of the central nervous system.

Among different methods to measure intelligence, Raven's progressive matrices [24] and analogous tests are considered to be closest to the “g” factor of intelligence, a concept used to describe the common underlying component of different intellectual abilities [17]. Such tests show stronger correlations with brain volume than other types of tests [32] and are tightly correlated with neurophysiological measures [17] reflecting the speed and reliability of neural transmission and the integrity of central nervous system. Our finding that stroke was predicted exclusively by low scores in this type of test, in particular in relation to the two other subtests, whereas coronary heart disease was equally predicted by low scores assessing different abilities, suggests that the association of intelligence with stroke is likely to be mediated by mechanisms related to the integrity of the central nervous system rather than by mechanisms related to common risk factors of stroke and coronary heart disease such as hypertension and atherosclerosis. Consistent with this, the visuospatial reasoning score of the Finnish Defence Forces test is a strong predictor of another disorder or the central nervous system, schizophrenia, whereas the other two scores are not [23].

Intellectual abilities and stroke could have common underlying risk factors operating during early life, possibly during fetal development. For example, a meta-analysis of 212 studies showed that, although heritability of intellectual abilities was high (around 48%), fetal environment accounted for 20% of the correlation of intelligence between identical twins [33]. In the present cohort, lower visuospatial abilities are predicted by thinness and small head circumference at birth [16]. In the whole source cohort of 13,345 individuals, stroke was also predicted by thinness at birth, although not by head circumference [34].

Men of this cohort who grew up in families of low socio-economic status, or who as adults had low socio-economic status, had on average lower intellectual ability scores (including visuospatial reasoning) [1]. They also had higher rates of stroke [34]. However, the association we found between visuospatial reasoning in relation to the two other subscores and stroke remained strikingly similar after adjustment for multiple indicators of socio-economic status throughout life. Interaction analyses showed that this association was similar across all socio-economic groups in childhood and in adult life, with the exception that no association was present among men who attained the highest occupational status. We would, however, urge caution in interpreting this finding because of the relatively large number of interaction analysis made. Our findings are paralleled by a recent study showing that whereas low fluid and crystallized intelligence measured at age 70 both predict higher mortality rates, adjustment for socio-economic status attenuates the association with crystallized but not with fluid intelligence [35]. Together these findings again argue for the “body and central nervous system integrity hypothesis”. In addition, the association between stroke and indicators of early life events, such as exposure to maternal pre-eclampsia [36] or reduced early growth [34], are not affected by adjustment for socio-economic status. This suggests that socio-economic status is unlikely to be a significant common underlying factor or a mediator of the link between low visuospatial reasoning and stroke.

### Strengths and limitations

The strengths of this study include the intellectual ability test performed during compulsory military service in early adulthood and the near-comprehensive availability of later-life data through national registers. In validation studies the hospital and death registers show an over 85% agreement for coronary heart disease and stroke against individual hospital records [25], [26], [27], [28]. We have previously discussed the limitations of the Helsinki Birth Cohort Study [20]. The present study was limited to men. In addition, not all men in the HBCS underwent the intellectual ability test. Although a detailed non-participation analysis raised little concern over participation bias [1], the smaller number of participants resulted in decreased power to detect small to moderate effects. For example, while adjustment for socio-economic status produced little change in the main effects of the hazard ratios, the confidence intervals of visuospatial reasoning subscores predicting stroke overlap one after this adjustment, whereas those of visuospatial reasoning adjusted for the two other subscores remain statistically significant. Moreover, we lack data for some potential confounders such as smoking and use of alcohol. Whereas we have adjusted for blood pressure, a major risk factor for stroke, our data consist of systolic blood pressure measured during military service and special reimbursement for antihypertensive medication, which capture only a part of variation in blood pressure. People with untreated hypertension may be at a similar or even higher risk for stroke than those who receive medication.

### Conclusions

We found that coronary heart disease was equally predicted by low scores in subtests measuring different intellectual abilities, while stroke was predicted a profile characterized by lower visuospatial reasoning scores in relation to the two other subscores. The association with stroke was independent of socio-economic status in childhood and over the course of adult life. We suggest that the association with stroke may be mediated through a common underlying factor, possibly an early impairment in brain development.

## References

[1] Kajantie E, Räikkönen K, Henriksson M, Forsén T, Heinonen K, et al (2010) Childhood socio-economic status modifies the association between intellectual abilities at age 20 and mortality in late life. J Epidemiol Community Health 64: 963–969. 19822561 10.1136/jech.2009.086967 PMC2976067

[2] Batty GD, Wennerstad KM, Smith GD, Gunnell D, Deary IJ, et al (2009) IQ in early adulthood and mortality by middle age: cohort study of 1 million Swedish men. Epidemiology 20: 100–109. 19234402 10.1097/EDE.0b013e31818ba076

[3] Batty GD, Shipley MJ, Mortensen LH, Boyle SH, Barefoot J, et al (2008) IQ in late adolescence/early adulthood, risk factors in middle age and later all-cause mortality in men: the Vietnam Experience Study. J Epidemiol Community Health 62: 522–531. 18477751 10.1136/jech.2007.064881 PMC3650086

[4] Batty GD, Deary IJ, Gottfredson LS (2007) Premorbid (early life) IQ and later mortality risk: systematic review. Ann Epidemiol 17: 278–288. 17174570 10.1016/j.annepidem.2006.07.010

[5] Hart CL, Taylor MD, Davey Smith G, Whalley LJ, Starr JM, et al (2003) Childhood IQ, social class, deprivation, and their relationships with mortality and morbidity risk in later life: prospective observational study linking the Scottish Mental Survey 1932 and the Midspan studies. Psychosom Med 65: 877–883. 14508035 10.1097/01.psy.0000088584.82822.86

[6] O'Toole BI, Stankov L (1992) Ultimate validity of psychological tests. Person Individ Diff 13: 699–716.

[7] Whalley LJ, Deary IJ (2001) Longitudinal cohort study of childhood IQ and survival up to age 76. Bmj 322: 819. 11290633 10.1136/bmj.322.7290.819 PMC30556

[8] Hemmingsson T, v Essen J, Melin B, Allebeck P, Lundberg I (2007) The association between cognitive ability measured at ages 18–20 and coronary heart disease in middle age among men: a prospective study using the Swedish 1969 conscription cohort. Soc Sci Med 65: 1410–1419. 17582667 10.1016/j.socscimed.2007.05.006

[9] Batty GD, Mortensen EL, Nybo Andersen AM, Osler M (2005) Childhood intelligence in relation to adult coronary heart disease and stroke risk: evidence from a Danish birth cohort study. Paediatr Perinat Epidemiol 19: 452–459. 16269073 10.1111/j.1365-3016.2005.00671.x

[10] Lawlor DA, Batty GD, Clark H, McIntyre S, Leon DA (2008) Association of childhood intelligence with risk of coronary heart disease and stroke: findings from the Aberdeen Children of the 1950s cohort study. Eur J Epidemiol 23: 695–706. 18704700 10.1007/s10654-008-9281-z

[11] Batty GD, Shipley MJ, Mortensen LH, Gale CR, Deary IJ (2008) IQ in late adolescence/early adulthood, risk factors in middle-age and later coronary heart disease mortality in men: the Vietnam Experience Study. Eur J Cardiovasc Prev Rehabil 15: 359–361. 18525394 10.1097/HJR.0b013e3282f738a6

[12] Batty GD, Shipley MJ, Gale CR, Mortensen LH, Deary IJ (2008) Does IQ predict total and cardiovascular disease mortality as strongly as other risk factors? Comparison of effect estimates using the Vietnam Experience Study. Heart 94: 1541–1544. 18801778 10.1136/hrt.2008.149567 PMC2602751

[13] Silventoinen K, Modig-Wennerstad K, Tynelius P, Rasmussen F (2007) Association between intelligence and coronary heart disease mortality: a population-based cohort study of 682 361 Swedish men. Eur J Cardiovasc Prev Rehabil 14: 555–560. 17667647 10.1097/HJR.0b013e328014672e

[14] Hart CL, Taylor MD, Smith GD, Whalley LJ, Starr JM, et al (2004) Childhood IQ and cardiovascular disease in adulthood: prospective observational study linking the Scottish Mental Survey 1932 and the Midspan studies. Soc Sci Med 59: 2131–2138. 15351478 10.1016/j.socscimed.2004.03.016

[15] Modig Wennerstad K, Silventoinen K, Tynelius P, Bergman L, Rasmussen F (2010) Association between intelligence and type specific stroke: a population-based cohort study of early fatal and non-fatal stroke in one million Swedish men. J Epidemiol Community Health 64: 908–912. 19833609 10.1136/jech.2008.084020

[16] Räikkönen K, Forsén T, Henriksson M, Kajantie E, Heinonen K, et al (2009) Growth trajectories and intellectual abilities in young adulthood: The Helsinki Birth Cohort study. Am J Epidemiol 170: 447–455. 19528290 10.1093/aje/kwp132

[17] Gray JR, Thompson PM (2004) Neurobiology of intelligence: science and ethics. Nat Rev Neurosci 5: 471–482. 15152197 10.1038/nrn1405

[18] Shea DL, Lubinski D, Benbow CP (2001) Importance of assessing spatial ability in intellectually talented young adolescents: a 20-year follow-up study. J Education Psychol 93: 604–614.

[19] Rohde TE, Thompson LA (2007) Predicting academic achievement with cognitive ability. Intelligence 35: 83–92.

[20] Barker DJ, Osmond C, Forsén TJ, Kajantie E, Eriksson JG (2005) Trajectories of growth among children who have coronary events as adults. N Engl J Med 353: 1802–1809. 16251536 10.1056/NEJMoa044160

[21] Paile-Hyvärinen M, Kajantie E, Räikkönen K, Henriksson M, Leskinen JT, et al (2009) Intellectual ability in early adulthood and type 2 diabetes in later life. Acta Diabetol 46: 249–252. 19271118 10.1007/s00592-009-0102-y

[22] Tuovinen S, Räikkönen K, Kajantie E, Leskinen JT, Henriksson M, et al (2012) Hypertensive disorders in pregnancy and intellectual abilities in the offspring in young adulthood: The Helsinki Birth Cohort Study. Ann Med 44: 394–403. 21495787 10.3109/07853890.2011.573497

[23] Tiihonen J, Haukka J, Henriksson M, Cannon M, Kieseppä T, et al (2005) Premorbid intellectual functioning in bipolar disorder and schizophrenia: results from a cohort study of male conscripts. Am J Psychiatry 162: 1904–1910. 16199837 10.1176/appi.ajp.162.10.1904

[24] Raven J (2000) The raven's progressive matrices: change and stability over culture and time. Cognit Psychol 41: 1–48. 10945921 10.1006/cogp.1999.0735

[25] Tolonen H, Salomaa V, Torppa J, Sivenius J, Immonen-Räihä P, et al (2007) The validation of the Finnish Hospital Discharge Register and Causes of Death Register data on stroke diagnoses. Eur J Cardiovasc Prev Rehabil 14: 380–385. 17568236 10.1097/01.hjr.0000239466.26132.f2

[26] Pajunen P, Koukkunen H, Ketonen M, Jerkkola T, Immonen-Räihä P, et al (2005) The validity of the Finnish Hospital Discharge Register and Causes of Death Register data on coronary heart disease. Eur J Cardiovasc Prev Rehabil 12: 132–137. 15785298 10.1097/00149831-200504000-00007

[27] Leppälä JM, Virtamo J, Heinonen OP (1999) Validation of stroke diagnosis in the National Hospital Discharge Register and the Register of Causes of Death in Finland. Eur J Epidemiol 15: 155–160. 10204645 10.1023/a:1007504310431

[28] Rapola JM, Virtamo J, Korhonen P, Haapakoski J, Hartman AM, et al (1997) Validity of diagnoses of major coronary events in national registers of hospital diagnoses and deaths in Finland. Eur J Epidemiol 13: 133–138. 9084994 10.1023/a:1007380408729

[29] Eriksson J, Forsén T, Tuomilehto J, Osmond C, Barker D (2000) Fetal and childhood growth and hypertension in adult life. Hypertension 36: 790–794. 11082144 10.1161/01.hyp.36.5.790

[30] Deary IJ, Whalley LJ, Lemmon H, Crawford JR, Starr JM (2000) The stability of individual differences in mental ability from childhood to old age: Follow-up of the 1932 Scottish mental survey Intelligence. 28: 49–55.

[31] Batty GD, Shipley MJ, Dundas R, Macintyre S, Der G, et al (2009) Does IQ explain socio-economic differentials in total and cardiovascular disease mortality? Comparison with the explanatory power of traditional cardiovascular disease risk factors in the Vietnam Experience Study. Eur Heart J 30: 1903–1909. 19602715 10.1093/eurheartj/ehp254 PMC2719700

[32] MacLullich AM, Ferguson KJ, Deary IJ, Seckl JR, Starr JM, et al (2002) Intracranial capacity and brain volumes are associated with cognition in healthy elderly men. Neurology 59: 169–174. 12136052 10.1212/wnl.59.2.169

[33] Devlin B, Daniels M, Roeder K (1997) The heritability of IQ. Nature 388: 468–471. 9242404 10.1038/41319

[34] Osmond C, Kajantie E, Forsen TJ, Eriksson JG, Barker DJ (2007) Infant growth and stroke in adult life: the Helsinki birth cohort study. Stroke 38: 264–270. 17218608 10.1161/01.STR.0000254471.72186.03

[35] Batterham PJ, Christensen H, Mackinnon AJ (2009) Fluid intelligence is independently associated with all-cause mortality over 17 years in an elderly community sample: An investigation of potential mechanisms. Intelligence 37: 551–560.

[36] Kajantie E, Eriksson JG, Osmond C, Thornburg KL, Barker DJ (2009) Preeclampsia is associated with increased risk of stroke in the adult offspring: The Helsinki Birth Cohort Study. Stroke 40: 1176–1180. 19265049 10.1161/STROKEAHA.108.538025

